# Jottings

**Published:** 1910-02-19

**Authors:** 


### Jottings.

Sir Ernest Shackleton will preside at the annual court of governors of the Seamen's Hospital Society at Prince's Restaurant on Monday, March 7, at 4 o'clock.

The sixty-fifth anniversary dinner of the German Hospital, Dalston, will be held at the Whitehall Rooms on Thursday, May 19, when the Right Hon. Sir Frank Cavendish Lascelles has kindly consented to take the chair.

Princess Christian has appointed Tuesday, May 24, as the date on which she will open the bazaar in aid of the Hospital for Women, to be held in the new hospital buildings in Soho Square, W. The ceremony will take place at 2.30 p.m. The object of the bazaar is to raise £5,000 for the completion of the rebuilding. If this amount is raised, the King Edward's Hospital Fund will give £3,000.

At the annual meeting of the trusteees of the Manchester Royal Infirmary on February 11 a resolution was passed by a fairly large majority stating that it is not desirable to appoint women to resident medical and surgical posts at the infirmary. The motion was proposed by Sir William Cobbett (the chairman), seconded by the Dean of Manchester (Bishop Welldon), and supported by Mr. J. E. Platt (chairman of the medical board).

The Halifax Guardians have decided to accept a tender of £37 for a new clock in the turret at the hospital. A member of the Hospital Committee explained that there were a good many clocks at the hospital, all worked by electricity, and no two of them kept the same time. The turret clock had caused a good deal of bad language in the district, and he thought that if the Board could save men's souls by spending £37 on a new clock it would be worth while!

The annual general court of the Governors and subscribers of the Jews' Hospital and Orphan Asylum was held on February 6, Mr. Felix A. Davis presiding. Mr. Alphonse Joseph (treasurer), in submitting the statement of accounts, showed a loss of £5,342 13s. 8d. for the year. The Chairman said that notwithstanding their financial position, it was decided to open a new institution at Norwood. This had become possible by reason of a donation of £11,000 from Mrs. Arnold Gabriel.

The Schiff Home of Recovery for Surgical Convalescents, of which the Earl of Lytton is Chairman, is to be opened in May or June. Knowle Hill Park, Cobham, with forty-eight acres, has been purchased, and there will be accommodation for some seventy patients. Cases will be sent from seven of the principal London hospitals, and the average period of residence will be three weeks. Between £30,000 and £40,000 had been subscribed when the scheme was secured by the munificent gift of £100,000 from Mr. Ernest F. Schiff. Colonel J. W. Wray, of Guildford, has been appointed secretary-superintendent of the Home.

At the annual governors' meeting of the London Fever Hospital, the report stated that the scarlet fever cases numbered 397, and of these 355 were discharged cured; 34 remained under treatment at the close of the year and eight died—a mortality of 2.2 per cent. The diphtheria cases numbered 65, and the mortality from this disease was only 1.8 per cent. The rebuilding of the hospital, not yet completed, has almost absorbed the invested funds, and it is now dependent upon voluntary subscriptions and payments from patients. Last year the amount received from legacies was only £179, as against an average of £3,054 in the preceding ten years.

